# The roles of monkey M1 neuron classes in movement preparation and execution

**DOI:** 10.1152/jn.00892.2011

**Published:** 2013-05-22

**Authors:** Matthew T. Kaufman, Mark M. Churchland, Krishna V. Shenoy

**Affiliations:** ^1^Neurosciences Program, Stanford University, Stanford, California;; ^2^Department of Electrical Engineering, Stanford University, Stanford, California;; ^3^Department of Bioengineering, Stanford University, Stanford, California;; ^4^Department of Neurobiology, Stanford University, Stanford, California;; ^5^Cold Spring Harbor Laboratory, Cold Spring Harbor, New York; and; ^6^Department of Neuroscience, Grossman Center for the Statistics of Mind, David Mahoney Center for Brain and Behavior Research, Kavli Institute for Brain Science, Columbia University Medical Center, New York, New York

**Keywords:** motor cortex, interneurons, pyramidal cells, cell type, gating

## Abstract

The motor cortices exhibit substantial activity while preparing movements, yet the arm remains still during preparation. We investigated whether a subpopulation of presumed inhibitory neurons in primary motor cortex (M1) might be involved in “gating” motor output during preparation, while permitting output during movement. This hypothesis predicts a release of inhibition just before movement onset. In data from M1 of two monkeys, we did not find evidence for this hypothesis: few neurons exhibited a clear pause during movement, and these were at the tail end of a broad distribution. We then identified a subpopulation likely to be enriched for inhibitory interneurons, using their waveform shapes. We found that the firing rates of this subpopulation tended to increase during movement instead of decreasing as predicted by the M1 gating model. No clear subset that might implement an inhibitory gate was observed. Together with previous evidence against upstream inhibitory mechanisms in premotor cortex, this provides evidence against an inhibitory “gate” for motor output in cortex. Instead, it appears that some other mechanism must likely exist.

many neurons in both premotor cortex and primary motor cortex (M1) are active during movement preparation (Riehle and Requin 1989; Tanji and Evarts 1976; Weinrich and Wise 1982). Given that activity in these areas also causes movement, we seek to better understand how preparatory activity is prevented from inadvertently producing movement.

Preparatory activity appears to be attenuated in stages: premotor cortex exhibits strong preparatory activity; M1 exhibits substantial preparatory activity but less than premotor cortex (see, e.g., Riehle and Requin 1989); the spinal cord exhibits modest preparatory activity (Fetz et al. 2002; Prut and Fetz 1999); and the muscles typically exhibit essentially no change during preparation. It is thus commonly assumed that considerable gating occurs at each of these stages, both in cortex and in the spinal cord, to achieve this stepwise reduction of preparatory activity. Given that M1 neurons receive many synapses from premotor areas (Dum and Strick 2002), it would seem there must be some mechanism reducing preparatory activity in these M1 neurons while not altogether divorcing them from all those potential inputs. More generally, we ask how preparatory activity can be present in some neurons (both in premotor and primary motor areas) without prematurely impacting other neurons.

Preparatory activity is frequently studied with a delayed-reach task. When a monkey is cued regarding the path of an upcoming reach but required to withhold the movement until a go cue, preparatory activity is present during the delay in both dorsal premotor cortex (PMd) and M1 (Riehle and Requin 1989; Tanji and Evarts 1976; Weinrich and Wise 1982). Preparatory activity covaries with a variety of upcoming movement parameters (Churchland et al. 2006b; Godschalk et al. 1985; Hocherman and Wise 1991; Messier and Kalaska 2000; Riehle and Requin 1989), predicts reaction time (Churchland et al. 2006c; Riehle and Requin 1993), predicts movement variability (Churchland et al. 2006a), and, if disrupted, delays the movement (Churchland and Shenoy 2007). Yet activity in PMd and M1 is related not only to preparing movement but also to controlling movement itself. These areas exhibit robust activity during movement (Evarts 1966; Wise et al. 1986), microstimulation in either area is sufficient to evoke movement (Dum and Strick 2002; Leyton and Sherrington 1917; Weinrich and Wise 1982), and pharmacological reduction of inhibition seems to impair withholding of premature movements (Sawaguchi et al. 1996). Given the preponderance of evidence that premotor and M1 activity is involved in both preparing and executing movements, and that many of M1's inputs are more active during preparation than M1 itself, theoretical models have posited a “gate” that can prevent preparatory activity from driving movement (e.g., Bullock and Grossberg 1988; Cisek 2006a), possibly via inhibition (Benjamin et al. 2010). Such a gate could allow some M1 neurons (those impacted by the gate) to remain relatively quiet during preparation even as their inputs (from other areas and from within M1) become active.

Building on recent work (Kaufman et al. 2010), here we further investigated possible gating mechanisms. Specifically, we examined the possibility that the relationship between activity in premotor areas and M1 might involve a nonlinear threshold or gating via time-varying inhibition.

The notion of a gate or threshold that keeps preparatory activity from driving movement has an obvious appeal, yet three lines of evidence are inconsistent with simple versions of a threshold model. First, preparatory activity is tuned very differently from movement-related activity in both M1 and PMd (Churchland et al. 2010; Crammond and Kalaska 2000; Kaufman et al. 2010). This is inconsistent with the notion that preparatory activity is a subthreshold version of movement activity. Second, higher firing rates do not translate into shorter reaction times (Churchland et al. 2006c; Crammond and Kalaska 2000). Again, these data are inconsistent with the idea that movements are generated when preparatory activity rises past a threshold. Finally, and most importantly, we recently sought evidence for an inhibitory gating mechanism in PMd and failed to find the predicted patterns of neural activity (Kaufman et al. 2010).

As noted in that study, however, there is at least one more simple possibility, that inhibition within M1 suppresses activity during preparation. In this view, the inputs arriving from premotor or other areas would recruit local inhibition within M1 during the preparatory period. This inhibition would then be released during movement generation. Consistent with this hypothesis, feedforward projections from PMd to inhibitory neurons in M1 have previously been found anatomically (Keller and Asanuma 1993) and physiologically (Ghosh and Porter 1988; Tokuno and Nambu 2000). Evidence against a related model in rats has been presented more recently (Isomura et al. 2009), but rats do not have a well-defined premotor-M1 separation and have only weakly tuned interneurons, in contrast to monkeys (Merchant et al. 2008). More generally, one suspects that there could be a variety of mechanisms/inputs that recruit gating inhibition within M1. It thus seems worth testing for such an effect directly, by looking to see whether there is a population of M1 neurons that exhibit gatelike responses. We therefore tested this “M1 gating model” in reaching monkeys by searching for gatelike neurons in M1. Specifically, we wished to know whether inhibition might fall around movement onset, allowing movement-causing activity to escape M1. We did not find a separate group of neurons in our recordings that paused during movement. This was true even for a population of neurons with narrow spike waveforms, which is likely to be substantially enriched for inhibitory neurons. These data thus argue that the inhibitory gating model is unlikely to be correct in M1. It would therefore seem that some other mechanism is responsible for attenuating preparatory activity from premotor areas to M1 to the spinal cord and muscles.

## MATERIALS AND METHODS

### 

#### Subjects.

Animal protocols were approved by the Stanford University Institutional Animal Care and Use Committee. The subjects were two adult male macaque monkeys (*Macaca mulatta*) trained to perform a variant of the delayed-reach task for juice reward. After initial training, we performed a sterile surgery during which the monkeys were implanted with a head restraint and a standard recording cylinder. The cylinders (Crist Instruments, Hagerstown, MD) were centered over the PMd/M1 border, initially estimated with stereotaxic coordinates (11–12 mm anterior to stereotaxic zero, the intermeatal “ear bar” line) and from previous surgeries and MRIs in other monkeys. In both cases the dura was reflected during a later surgery and the sulcal landmarks were directly visualized, confirming the previous stereotaxic coordinates. The cylinders were placed surface normal to the skull, which was left intact and covered with a thin layer of dental acrylic. To accommodate recording, 3-mm holes were drilled later under ketamine-xylazine anesthesia.

#### Task apparatus.

The task apparatus has been described previously (Churchland et al. 2006c). Briefly, during experiments monkeys sat in a customized chair (Crist Instruments) with the head and left arm restrained. Stimuli were back projected onto a frontoparallel screen ∼25 cm from the eyes. A photodiode was used to record the timing of video frames with 1-ms resolution. The position of an infrared-reflective bead taped to the fingers was tracked optically in the infrared (Polaris system; Northern Digital, Waterloo, ON, Canada). The eyes were also tracked in the infrared (Iscan, Burlington, MA). A tube dispensed juice rewards.

#### Task design.

**Fig. 1.**
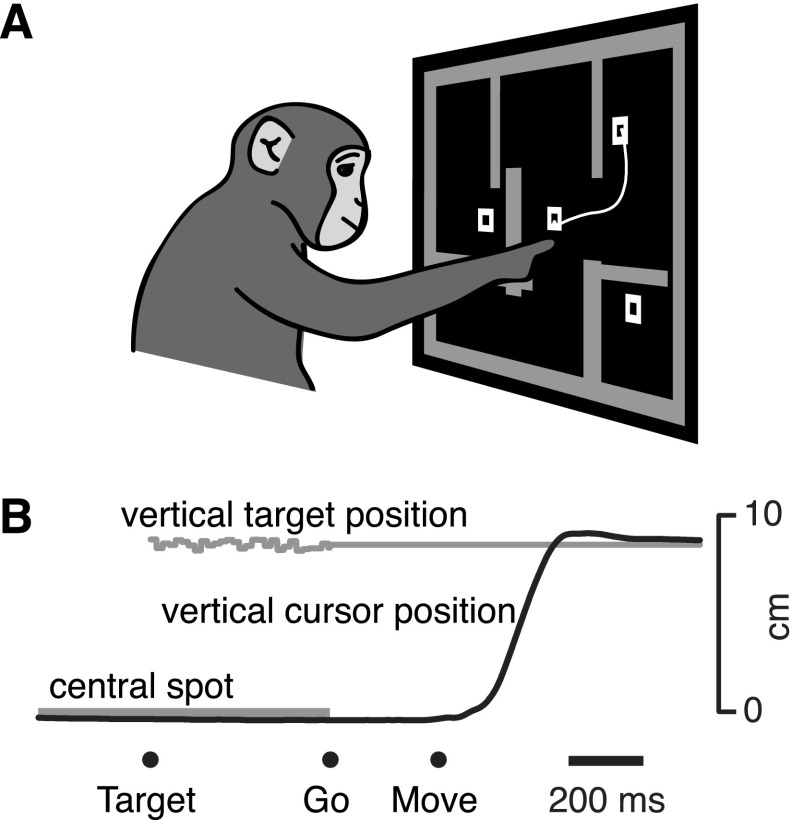
Behavioral task. *A*: the maze task. One of the many possible mazes is shown. *B*: a timeline of the task. The monkey initially touched a central spot, and then a target and (typically) a set of barriers appeared. On some trials, 2 inaccessible distracter “targets” appeared as well. The target(s) initially jittered slightly in place. The “go” cue was indicated by cessation of target jitter, the targets filling in, and the disappearance of the central spot. The monkey then had to make a curved reach around the barriers to touch the accessible target. Times are indicated at *bottom*. Target, target onset; Go, go cue; Move, movement onset. *Maze ID1* is shown.

Both monkeys performed a variant of the center-out delayed-reach task, called the “maze” task (Fig. 1), similar to that described previously (Kaufman et al. 2010). Here the maze task is used simply as a 27-condition (*monkey N*) or 108-condition (*monkey J*) delayed-reach task, but details are given below for completeness.

Experiments consisted of trials, each a few seconds long, that ended in a juice reward if successful. The animal controlled a cursor projected on the screen, offset a few centimeters above his optically tracked hand position. He began a trial by fixating (for at least 700 ms) a central fixation spot with his eyes while touching the spot with the cursor. On one-third of trials, a single target appeared. On another one-third of trials, a target and up to nine rectangular barriers appeared. The last one-third of trials was identical to the previous type, but an additional two distracter targets appeared as well (Fig. 1*A*). After a randomized preparatory period (0–1,000 ms), a go cue was given, and reaches were rewarded if they were swift and did not pass through a barrier. Reward was delivered after the target was held for 450 ms (*monkey J*) or 700 ms (*monkey N*), with the next trial beginning a few hundred milliseconds later. When the targets first appeared, they were hollow and jittered slightly (2–3 mm). The go cue was indicated by cessation of target jitter, the targets filling in, and the extinguishing of the fixation spot. A variety of unanalyzed catch trials were also interleaved, including randomly generated novel mazes. Reach curvature and other such parameters were not directly analyzed here. From the standpoint of the analyses to follow, the challenging nature of this task is largely irrelevant. The important feature of this task is that it evoked a variety of different reach types (left, right, straight, curved, near, far) and produced strong preparatory neural responses during the delay period.

#### Neural recordings.

Neural recordings were made with previously described techniques (Churchland et al. 2006c; Kaufman et al. 2010). Briefly, recordings were made one at a time with moveable tungsten single electrodes (Frederick Haer, Bowdoinham, ME) and a Plexon Multichannel Acquisition Processor (Plexon, Dallas, TX). Neurons were carefully isolated and discarded if more than a very rare refractory period violation was observed (i.e., >1 every several minutes; typically no violations at all were observed).

While the M1/PMd boundary cannot be identified definitively without histology, *monkey J*'s M1 recordings were located entirely within 5 mm anterior of the central sulcus, which has previously been described as M1 proper (Boudrias et al. 2010). For *monkey N*, a few recordings were as far as 6 mm anterior of the central sulcus and thus likely in the M1/PMd “transition zone” (Keller 1993; Weinrich and Wise 1982; Wise et al. 1986), but these recordings yielded results similar to those posterior when analyzed separately. For both monkeys, many of these recordings were made deep in the sulcus. Microstimulation at multiple sites in both monkeys evoked movements of the shoulder and upper arm or (much less often) of the wrist. Microstimulation thresholds were often 25 μA or less and as low as 3 μA.

#### Classification of neuron types.

We classified the waveforms as narrow or broad spiking on the basis of their shapes, using previously described methods (Kaufman et al. 2010; see below for discussion of these methods). In both monkeys, when we had a choice of two units to isolate, we preferentially isolated the unit with the narrower spike. This was done to increase the yield of narrow-spiking neurons, which were the focus of several of our analyses. However, to be conservative, precise measurements of spike width were not performed until after recordings were completed and we never discarded a well-isolated unit because of its spike width. Thus our preference for narrower spikes increased the number of narrow-spiking neurons recorded but is very unlikely to have influenced the bimodal nature of the distribution (we have seen the same bimodal distribution in previous recordings even when we showed no preference for narrow spikes; Kaufman et al. 2010).

Previous work has found that inhibitory interneurons generally exhibit spike waveforms that have a shape slightly different from that of pyramidal cells. In particular, these two groups of neurons have somewhat different distributions of the trough-to-peak duration (TTP) of the spike waveform. This difference has been found in several cortical areas of both rodent (Bartho et al. 2004; Isomura et al. 2009) and monkey (Gonzalez-Burgos et al. 2005; Krimer et al. 2005; Merchant et al. 2008). The combined distribution of TTPs is often bimodal, with the briefer mode thought to correspond with inhibitory interneurons and the longer mode containing mostly pyramidal neurons.

This metric appears to perform reasonably well but is not perfect. Inhibitory interneurons are a heterogeneous group (Kawaguchi and Kubota 1997; Markram et al. 2004), and some inhibitory interneurons have intermediate (Brill and Huguenard 2009; Gonzalez-Burgos et al. 2005; Krimer et al. 2005) or even broad (Merchant et al. 2008) waveforms. The distributions of spike width may also overlap slightly (for review, see Merchant et al. 2012). It has previously been estimated that ∼14% of inhibitory neurons in M1 are not narrow spiking (Merchant et al. 2008). Additionally, another previous study found that ∼3–5% of pyramidal tract neurons can exhibit spike waveforms as narrow as those of inhibitory neurons (Fig. 5 of Vigneswaran et al. 2011, using our 200-μs cutoff). It is also possible that other large-axon pyramidal cells (such as neurons projecting to subcortical or other cortical areas) may produce narrow action potentials or that use of different unit selection criteria may result in a fraction of pyramidal tract neurons that is different because of oversampling of large neurons (Humphrey and Corrie 1978; Towe and Harding 1970).

Despite these complications, when a bimodal distribution of TTPs can be found we expect that the narrow-spiking population will include ∼80–90% of all inhibitory neurons and that the broad-spiking population will include most of the excitatory neurons. While not a substitute for direct identification of neuron type, this method produces one population of neurons that is likely to be substantially enriched for inhibitory neurons and another that is enriched for excitatory neurons. Spike width-based techniques have therefore been used previously by a number of researchers for this purpose (e.g., Diester and Nieder 2008; Johnston et al. 2009; Mitchell et al. 2007; Rao et al. 1999; Wilson et al. 1994), including in M1 (Merchant et al. 2008).

**Fig. 2.**
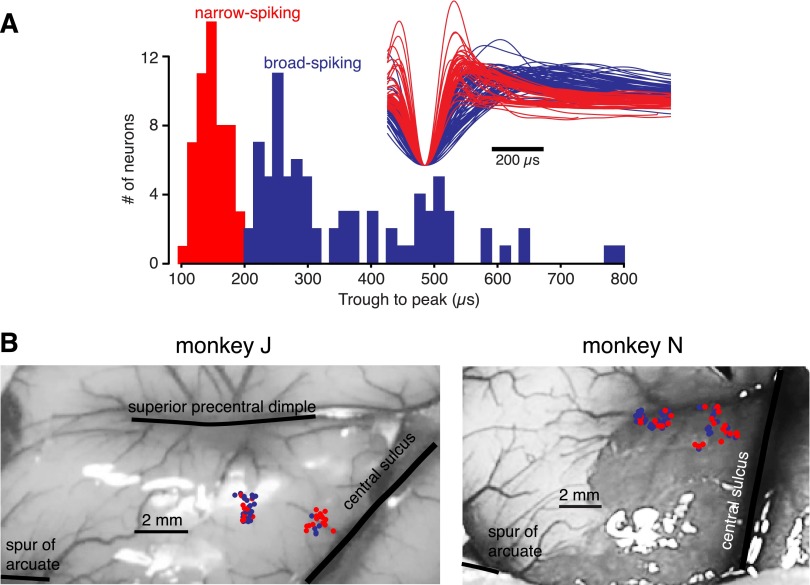
Recording locations and neuron classification. *A*: distribution of trough-to-peak durations (TTPs), combined across animals. *Inset*: mean waveforms from all classified M1 recordings. *B*: surface entry points of the recording locations are superimposed on a photograph of the monkey's brain, with major landmarks highlighted with thick black lines. Only classified units are shown. For *monkey J*, alignment is estimated based on within-recording cylinder measurements and positioning of the cylinder on the skull. For *monkey N*, recording sites were registered with a photograph of his brain using measurements taken intraoperatively. Note that recordings were performed at many depths, spanning a substantial fraction of this region of M1. Blue dots indicate recordings with broad waveforms; red dots indicate recordings with narrow waveforms. Dots are randomly displaced slightly (0.1 mm) to reveal overlapping recordings.

Recordings were made in the range of medio-lateral locations that best produced shoulder or upper arm movements when microstimulation was performed. Seventy-seven neurons were collected from *monkey J*; 93 were collected from *monkey N*. Recording locations of classified units are shown in Fig. 2*B*. Only the surface entry points of the electrode penetrations are shown; recordings were performed over a wide range of electrode depths, spanning the full depth of the sulcus. The median number of analyzed trials per neuron was 352 for *monkey J* and 301 for *monkey N* (13 and 11 trials per reach condition).

The cutoff for classifying neurons as narrow or broad spiking was chosen to be 200 μs, matching the apparent dip in the present data from M1 (see results) and the previously found dip in the TTP distribution in PMd when using identical methods (Kaufman et al. 2010). We note that the appropriate cutoff for other data sets is likely to vary depending on brain area, electrode type, filter settings, and perhaps other factors (Merchant et al. 2012; Vigneswaran et al. 2011). To test the TTP distributions for bimodality, we combined data across animals and used Hartigan's dip test with a bootstrap (Hartigan and Hartigan 1985; Mechler and Ringach 2002). Since this test is sensitive to skewed distributions (Jackson et al. 1989), we performed this test only on TTP values <300 μs, where we estimated the distribution to be approximately symmetrical (for all other analyses, all classifiable neurons were used).

#### EMG recordings.

EMG activity was recorded from both monkeys with hook-wire electrodes (44 gauge with a 27-gauge cannula; Nicolet Biomedical, Madison, WI) inserted into a muscle for single recording sessions, which were interleaved with the neural recording sessions. For *monkey J*, recordings were made sequentially from trapezius, latissimus dorsi, pectoralis, triceps brachii, medial and lateral aspects of the biceps brachii, and anterior, medial, and posterior aspects of the deltoid. For *monkey N*, recordings were made from proximal, middle, and distal aspects of the trapezius, latissimus dorsi, pectoralis, triceps brachii, medial and lateral aspects of the biceps, and anterior, medial, and posterior aspects of the deltoid. Electrode voltages were amplified, band-pass filtered (150–500 Hz, four pole, 24 db/octave), sampled at 1,000 Hz, and digitized. Off-line, raw traces were differentiated (to remove any remaining baseline), rectified, smoothed with a Gaussian (SD of 15 ms), and averaged.

We verified that no sizeable anticipatory changes were present in muscle activity during the preparatory epoch. EMG activity was typically unmodulated during preparation, or in rare cases very weakly modulated during preparation. This is consistent with previous verifications in prior, similar experiments (Churchland et al. 2006b, 2006c; Kaufman et al. 2010).

## RESULTS

### 

#### Separation of narrow- and broad-spiking neurons.

We made recordings from 170 neurons in two monkeys from surface and sulcal M1 (Fig. 2*B*; note that neurons were recorded both near the surface and in the sulcus below the entry sites shown). We found a bimodal distribution of TTP of the waveforms (Fig. 2*A*, monkeys pooled; no qualitative difference was observed between animals). To statistically confirm bimodality, we performed a Hartigan's dip test (Hartigan and Hartigan 1985; Mechler and Ringach 2002) with a bootstrap and 100,000 iterations. We first truncated the distributions at 300 μs, to reduce skewness, which could otherwise invalidate the test (Jackson et al. 1989). Bimodality was confirmed with *P* < 0.05 (*n* = 95).

In *monkey J*, 25 M1 neurons were classifiable as narrow spiking and 36 were classifiable as broad spiking. In *monkey N*, 27 M1 neurons were classifiable as narrow spiking and 44 were classifiable as broad spiking. Some recorded neurons were not classified either because their waveforms lacked a posttrough peak or because they exhibited a flattened posttrough peak that could not be measured reliably; only the neurons with classified waveforms are shown in Fig. 2. The fraction of our recordings identified as narrow spiking (41% for *monkey J*; 38% for *monkey N*) was greater than physiological (20–30%; Connors and Gutnick 1990), likely because an effort was made to preferentially isolate neurons with waveforms that appeared to be narrow (see materials and methods). In previous experiments in which we did not preferentially isolate narrow-spiking neurons, we obtained a fraction of narrow-spiking neurons similar to the fraction present anatomically (Kaufman et al. 2010). No analyses showed relevant differences when neurons were analyzed separately based on their depths or anterior-posterior locations.

#### Testing the M1 gating model.

**Fig. 3.**
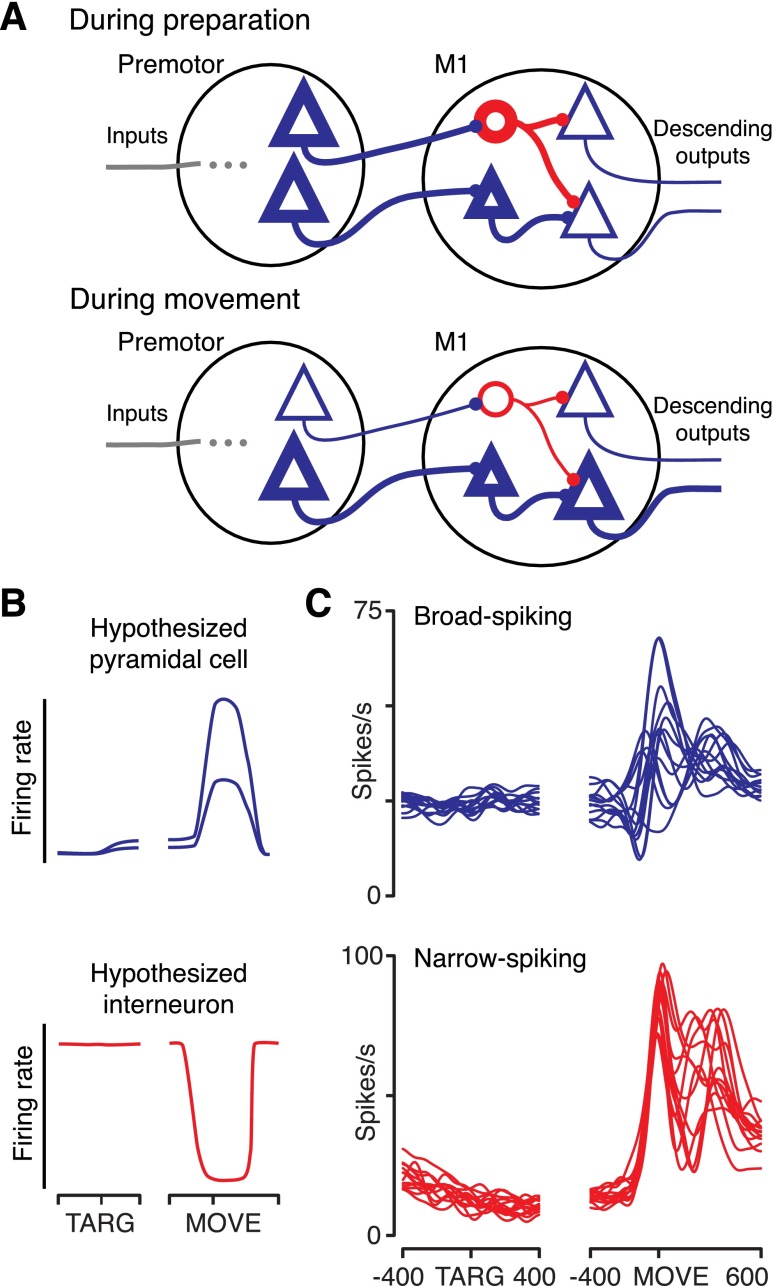
M1 gating model and example recordings. Heavy outlines in illustrations represent active neurons. In this model (*A*), most M1 neurons are not very active during motor preparation (*top*) because of strong inhibition within M1 during the preparatory epoch. During movement (*bottom*), this internal inhibition declines and premotor activity (possibly from numerous areas) drives M1 activity. *B*: responses predicted by the model. Pyramidal cells are expected to be more active during movement than during preparation, and interneurons are expected to have high firing rates during baseline and preparation and then pause during movement. *C*: peristimulus time histograms (PSTHs) of a recorded broad-spiking (putative pyramidal) neuron from M1 (*top*) and of a narrow-spiking (putative inhibitory interneuron) neuron from M1 (*bottom*). Both examples were selected to be as representative as possible. Half of the conditions were selected randomly for display, to aid clarity. PSTHs were smoothed with a 30-ms Gaussian. *Neurons J-PM167* and *J-PM206* are represented.

We previously reported evidence that was not consistent with the hypothesis that inhibition native to PMd suppresses output during movement preparation (Kaufman et al. 2010). This implies that PMd, and likely other areas as well, should drive M1 during movement preparation. It would therefore seem that some mechanism is needed to attenuate these inputs to M1 and prevent premature movements. Here we consider the M1 gating model, as illustrated in Fig. 3*A*. In this model premotor areas activate inhibitory neurons within M1 during preparation, preventing M1 output. These premotor areas might include PMd or supplementary motor areas, or perhaps subcortical areas as well. During movement, inhibition within M1 is released, permitting M1 to become more active. The M1 gating model makes specific predictions about the pattern of firing rates that should be observed in both pyramidal neurons and inhibitory interneurons in M1 (Fig. 3*B*). Most importantly, M1 interneurons would be expected to have high tonic firing rates during both baseline and preparation and then pause during movement. Most pyramidal neurons should, in contrast, tend to be more active during movement than during preparation.

There are a number of plausible variants of this model. For example, perhaps local inhibition is primarily recruited by a subset of neurons local to M1. In many such models the key feature is that local inhibition within M1 should be high during preparation and lower during movement.

Peristimulus time histograms (PSTHs) from representative broad-spiking and narrow-spiking neurons are shown in Fig. 3*C*. Both example neurons exhibit complex, time-varying responses during the movement, and these responses are substantially different for different reach conditions (individual traces). While the broad-spiking neuron (a putative pyramidal neuron) arguably resembles the pattern expected from the model, the narrow-spiking neuron (a putative inhibitory interneuron) exhibits a pattern of activity nearly the opposite of what was expected from the model. Instead of having a high baseline rate and then pausing during movement, this neuron has a low baseline firing rate and then increases its response for all conditions during movement.

**Fig. 4.**
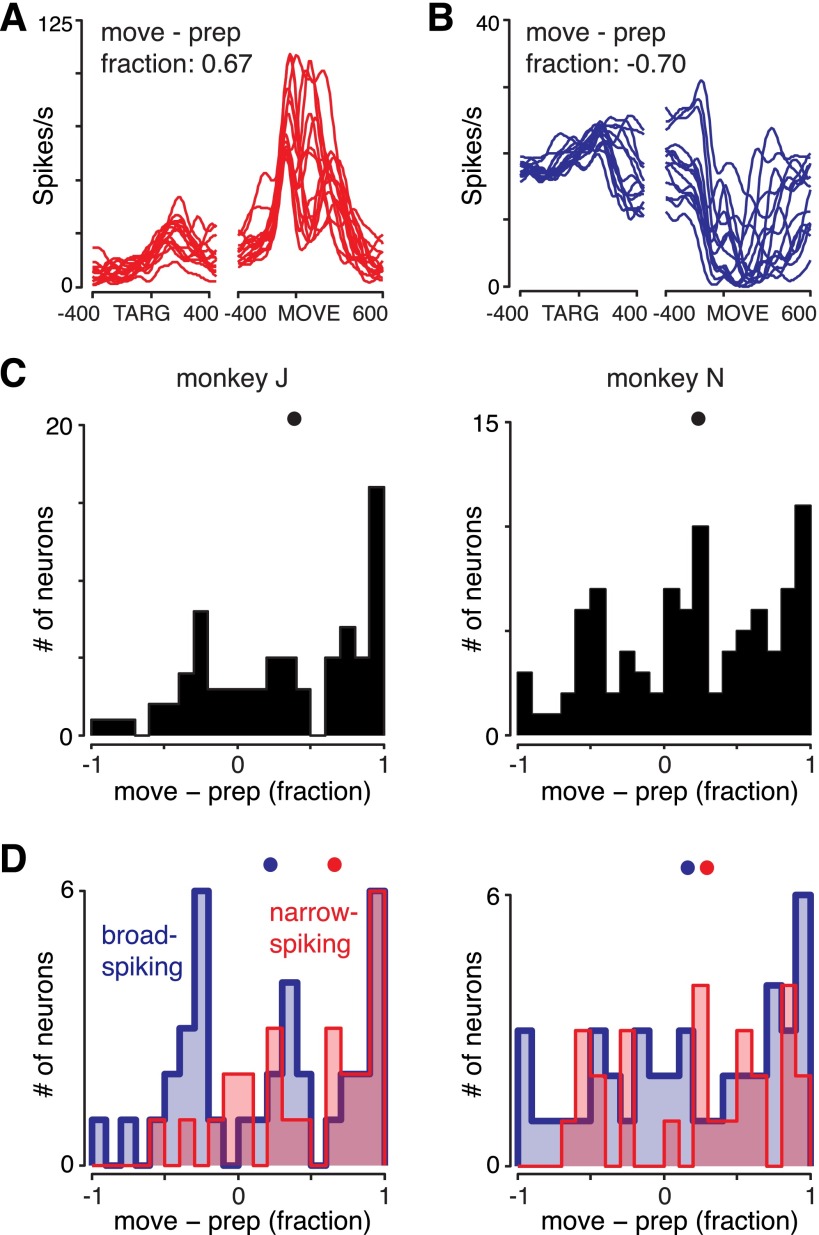
Cell-by-cell analysis of movement-epoch activity relative to preparatory activity. For each neuron, the mean firing rate was taken during a portion of the movement (−100 to +200 ms from movement onset), the mean firing rate during preparation was subtracted (50–400 ms after target onset), and the result was normalized by whichever of these 2 values was larger. A neuron that is completely silent around movement thus has an index of −1. *A* and *B*: PSTHs for 2 example neurons, with their indexes of movement activity. *A*: a narrow-spiking neuron with a positive index (*neuron J-PM125*). *B*: a broad-spiking neuron with a negative index (*neuron J-PM98*). *C*: histogram of the movement indexes for *monkey J* (*left*) and *monkey N* (*right*) across all neurons. Black dots show medians. While some units shut off during movement, these appear to form the tail of a broad distribution. *D*: histograms of the same index, with units segregated based on waveform shape. Red indicates narrow-spiking neurons; blue indicates broad-spiking neurons. Dots indicate medians. The narrow-spiking units do not appear to be more gatelike than the broad-spiking units.

Since the M1 gating hypothesis predicts the existence of inhibitory neurons with high firing rates during preparation and low rates during movement, we searched for a population of neurons that paused consistently during movement. For each neuron, we computed a movement activity index of movement activity (averaged from −100 to +200 ms from movement onset) relative to preparatory activity (averaged from 50 to 400 ms after target onset). The index was simply the firing rate during movement minus the firing rate during preparation, normalized by whichever of the two values was greater. This index is bounded from −1 to +1. If the index is negative, the neuron tended to pause during movement. PSTHs of two more example neurons, with their corresponding indexes, are shown in Fig. 4, *A* and *B*.

We initially concentrated on the distribution over all neurons (broad spiking, narrow spiking, and unclassifiable) to ask whether there was any subset of neurons with clear pauses during movement. The distribution of the movement activity index is shown in Fig. 4*C*. The distributions are relatively broad, with more values near 1 (neurons only active during movement) and with relatively few neurons having indexes near −1 (pausing during movement). Only a small percentage of neurons showed a tendency to pause during movement (6% of neurons had an index < −0.5 for *monkey J*, 14% for *monkey N*), and this pausing tendency was rarely complete. For example, the neuron in Fig. 4*B* has an index of −0.7. While this did indeed reflect a tendency to pause on average, this tendency was incomplete and varied across conditions; this neuron still showed substantial structured movement activity. In summary, if inhibitory gating is present, the signal appears to be carried by a very small subset of neurons.

A reasonable question is thus whether the few neurons that do pause during movement are more likely to be inhibitory interneurons as opposed to pyramidal neurons. Classifying neurons using waveform shape alone does not provide perfect identification of inhibitory and excitatory neurons (see materials and methods), yet the narrow-spiking population is likely to be substantially enriched for inhibitory neurons while the broad-spiking population is likely to be substantially enriched for excitatory neurons. One can thus ask whether those neurons that do pause during movement tend to be narrow spiking. We found the reverse to be true: narrow-spiking units are if anything less likely to pause during movement than broad-spiking units (Fig. 4*D*; not significant for either monkey, Mann-Whitney *U*-test). Thus, even in a subset of neurons likely to be enriched for inhibitory units, no population of “gatelike” neurons is obvious.

**Fig. 5.**
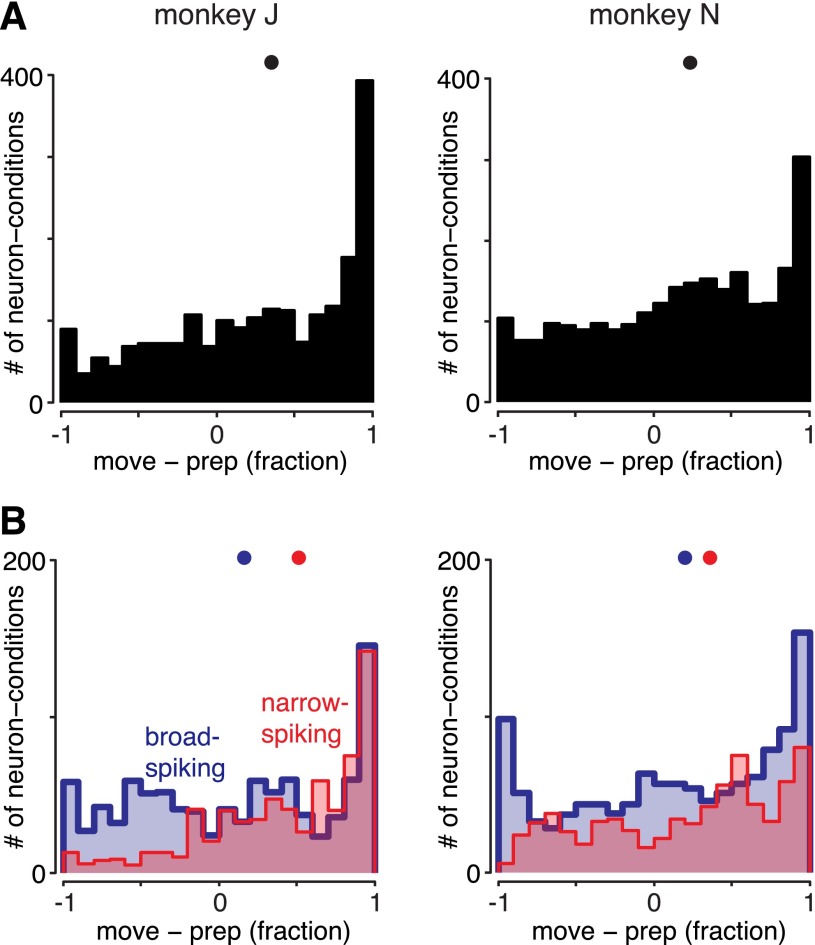
Analysis of movement-epoch activity relative to preparatory activity, by neuron and condition. This figure is the same as Fig. 4, *C* and *D*, but there conditions (reach shapes) were averaged to obtain 1 sample per neuron. Here each condition for each neuron is a sample. Thus if some neurons shut off during movement only for some conditions, they should be apparent. *A*: no separate group of such neuron-conditions is obvious. *B*: narrow-spiking units (red) are again less likely than broad-spiking units (blue) to shut off during movement.

**Fig. 6.**
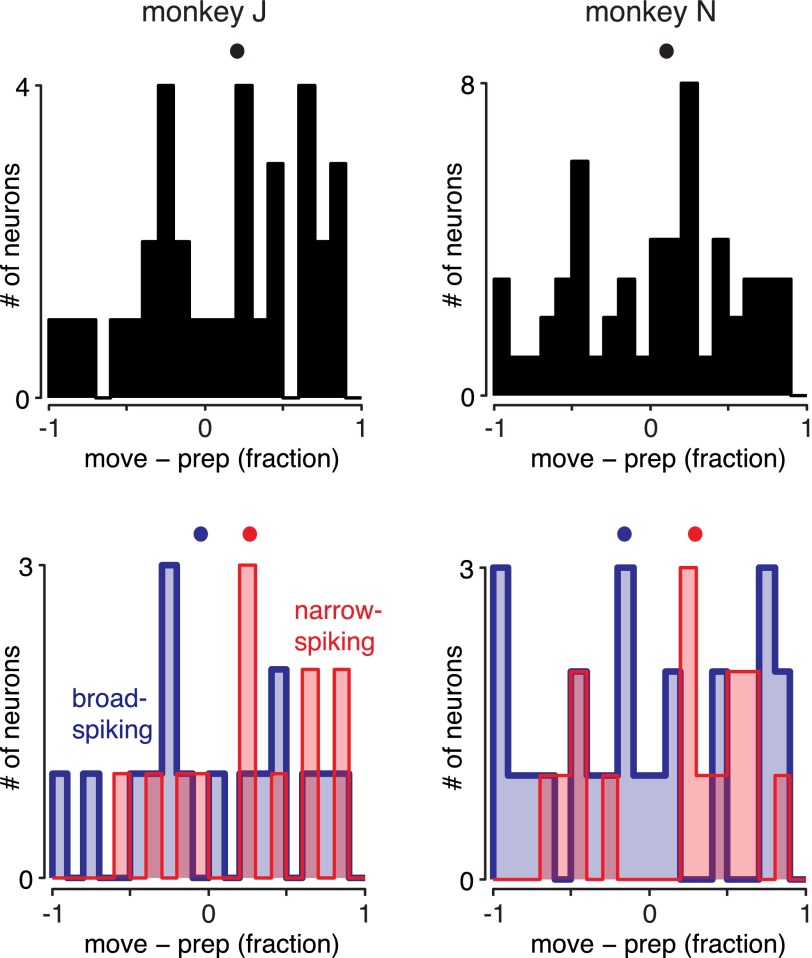
Cell-by-cell analysis of firing rates for preparatory-tuned neurons only. This figure is the same as Fig. 4, *C* and *D*, but includes only neurons whose preparatory tuning was at least 8 spikes/s. Tuning was assessed by taking the average for each condition from 100 to 400 ms after target onset and then taking the range across conditions. Distributions for *monkey J* (*left*) and *monkey N* (*right*) are shown. Most importantly, as in Fig. 4, the distributions of the movement activity index show few neurons that tend to pause (few neurons having indexes near −1), and those neurons that do tend to pause appear to be part of a broad distribution.

As an alternative, it is possible that inhibitory neurons pause for some conditions but not others. To test this, we can examine the distribution of movement activity across neurons and conditions, instead of averaging over conditions as above. There is thus one sample per neuron per condition (each condition being a reach of a particular type). Again, we did not find an excess of values near −1 (Fig. 5*A*). When neurons are segregated by spike width, narrow-spiking units are again less likely than broad-spiking units to pause during movement (Fig. 5*B*; *P* < 0.001 for *monkey J*, not significant for *monkey N*). Results were also similar when only preparatory-tuned neurons were examined (Fig. 6).

## DISCUSSION

Models of motor preparation often tacitly assume a gating mechanism: one that prevents preparatory activity from prematurely cascading through the system and reaching the muscles. Here, we tested the M1 gating model, which posits that inhibition within M1 keeps neurons from responding inappropriately during the delay. We found that the properties of M1 neurons, including putative inhibitory interneurons, provided little support for this model.

The M1 gating hypothesis illustrated in Fig. 3*A* makes a clear prediction: activity of inhibitory neurons should on average be high during the preparatory period and low during the movement period. We did not find any separate subpopulation of such neurons, and the activity of narrow-spiking neurons (putative interneurons) tended to be highest, not lowest, around the time of the movement. Previous work has found a similar pattern in narrow-spiking neurons in PMd (Kaufman et al. 2010), providing evidence against gating of output there as well. Studies have also provided evidence against use of a nonlinear threshold: preparatory activity does not appear to be a subthreshold version of movement activity (Churchland et al. 2006c, 2010; Crammond and Kalaska 2000; Kaufman et al. 2010).

Our data also suggest that rising excitation “breaking through” inhibition is not likely, since narrow-spiking neurons were at least as likely as broad-spiking neurons to exhibit a rise in firing rate during movement. However, testing this hypothesis directly would require identification of input and output neurons, which was not done here.

Some explanation for how preparatory activity is reduced at each stage is therefore still required (Green and Kalaska 2011). Although simple inhibitory gating and threshold models now appear unlikely, several other possibilities exist.

### 

#### Suppression of preparatory activity at multiple stages.

The suppression of preparatory activity in the motor system appears to occur in stages, with preparatory activity being strongest in premotor cortex, weaker in M1, weaker still in the spinal cord, and virtually absent in the muscles. Mechanisms within the spinal cord can obviously account for only some of these observations, but below we review the evidence that there are spinal mechanisms that limit the ability of preparatory activity to impact muscle activity (Moll and Kuypers 1977).

A sizeable fraction of pyramidal tract neurons are known to be active during preparation (Tanji and Evarts 1976). Some of this activity likely exists to set up key reflexes, perhaps so that the movement may be more quickly permitted when the time comes (Pruszynski et al. 2011; Selen et al. 2012) and/or so that other preparatory activity does not itself trigger movement (Duque and Ivry 2009; Fetz et al. 2002). These reflexes appear to be modulated in opposition to visually observed actions (Baldissera et al. 2001), which may be to counter “mirror” activity in premotor cortex (Fadiga et al. 1995; Rizzolatti and Craighero 2004) or internal preparation. This could be a form of gating: setting up reflexes to counter direct effects of preparatory activity.

Other forms of spinal gating may also be at work. Corticospinal excitability appears to be reduced selectively in the arm one is preparing to move (Duque and Ivry 2009), although this observation does not localize the mechanism as cortical or spinal. Furthermore, inhibition appears to rise in the spinal cord during preparation, and, while apparently modest, this inhibition is broadly tuned (Fetz et al. 2002; Prut and Fetz 1999). Thus it remains quite possible that inhibition contributed by the spinal cord plays a critical role in the control of preparatory activity.

In summary, the loss of preparatory activity occurs in stages. The exact mechanisms involved are not yet clear; nor is it clear that they are the same mechanisms at every stage. Inhibitory gating may be important in the spinal cord, but we found little evidence that such a mechanism is prevalent in M1.

#### Alternative models for controlling movement onset.

It is certainly possible that there exists an undiscovered or sparsely recorded set of interneurons that act as inhibitory gates. If this subclass of inhibitory neurons existed but were rare or small and difficult to record from, it could explain why our recordings did not sample them (or sampled few of them). Similarly, it might be that only a small subset of inhibitory neurons function as gates for any given task, and that the neurons recorded here might thus behave differently in a different task. Future studies using a greater range of behaviors, identified projection neurons (or input or pyramidal tract neurons), or spike-triggered EMG averages, would be needed to determine whether such a class might exist.

As suggested recently, there is another alternative, that no explicit gating mechanism is required (Kaufman et al. 2010). Taking the view that motor and premotor cortex comprise a dynamical system (Afshar et al. 2011; Churchland et al. 2006b, 2010; Cisek 2006b; Fetz 1992; Scott 2008; Shenoy et al. 2011, 2013; Todorov and Jordan 2002), one can more directly consider the relationship of neural activity in one brain area to activity in an area it projects to (e.g., PMd to M1). It seems likely that not all linear combinations of neurons would affect the downstream area—that is, many different patterns of PMd activity would evoke exactly the same M1 activity. Put mathematically, this is to say that many patterns of PMd activity would lie in the “null space” of M1. If preparatory activity were confined to such a null space, no further gating mechanism would be required. It could also explain the observed near-zero correlation between preparatory and movement-epoch neural activity (Churchland et al. 2010; Crammond and Kalaska 2000; Kaufman et al. 2010). Low correlations are expected under this model because the movement-period activity must (in order to generate movement) differ from preparatory activity in ways other than sheer magnitude. Yet, while consistent with known results, the concept of a “null space” clearly requires direct testing based on further predictions of the model.

In summary, we examined neurons in M1 to ask whether there might be an inhibitory gate for movement. We observed activity patterns that were inconsistent with those predicted by the gating hypothesis and were unable to find a distinct subset of neurons that were consistent with inhibitory gating. This was true even in a population identified by spike waveform shape as likely to be enriched for inhibitory neurons. Together with similar findings in PMd, this provides evidence that widespread inhibitory gating within cortex is not likely to be a major mechanism for preventing preparatory activity from flowing downstream. These results thus further narrow the space of possible mechanisms by which preparatory activity may avoid causing premature movements.

## GRANTS

This work was supported by a National Science Foundation graduate research fellowship (M. T. Kaufman), Burroughs Wellcome Fund Career Awards in the Biomedical Sciences (M. M. Churchland, K. V. Shenoy), the Christopher and Dana Reeve Foundation (K. V. Shenoy), National Institutes of Health (NIH) CRCNS R01-NS-054283 (K. V. Shenoy), NIH Director's Pioneer Award 1DP1OD006409 (K. V. Shenoy), and DARPA REPAIR N66001-10-C-2010 (K. V. Shenoy).

## DISCLOSURES

No conflicts of interest, financial or otherwise, are declared by the author(s).

## AUTHOR CONTRIBUTIONS

Author contributions: M.T.K., M.M.C., and K.V.S. conception and design of research; M.T.K. and M.M.C. performed experiments; M.T.K. analyzed data; M.T.K., M.M.C., and K.V.S. interpreted results of experiments; M.T.K. prepared figures; M.T.K. drafted manuscript; M.T.K., M.M.C., and K.V.S. edited and revised manuscript; M.T.K., M.M.C., and K.V.S. approved final version of manuscript.
